# Leukocidin genes *lukF-P83* and *lukM* are associated with *taphylococcus aureus* clonal complexes 151, 479 and 133 isolated from bovine udder infections in Thuringia, Germany 

**DOI:** 10.1186/1297-9716-43-42

**Published:** 2012-05-15

**Authors:** Katharina Schlotter, Ralf Ehricht, Helmut Hotzel, Martin Pfeffer, Karsten Donat

**Affiliations:** 1 Thüringer Tierseuchenkasse (Livestock Health Service & Insurance Agency of the State of Thuringia), Victor-Goerttler-Straße 4, 07745, Jena, Germany; 2 Alere Technologies GmbH, Loebstedter Straße 103-105, 07749, Jena, Germany; 3 Friedrich-Loeffler-Institut, Institute of Bacterial Infections and Zoonoses, Naumburger Straße 96a, 07743, Jena, Germany; 4 Institute for Medical Microbiology and Hygiene, Faculty of Medicine Carl Gustav Carus, Technical University of Dresden, Fiedlerstraße 42, 01307, Dresden, Germany; 5 Institute of Animal Hygiene and Veterinary Public Health, Centre of Veterinary Public Health, University Leipzig, An den Tierkliniken 1, 04103, Leipzig, Germany

## Abstract

*Staphylococcus aureus* is one of the most important causal agents of bovine mastitis. Population studies on bovine *Staphylococcus aureus* isolates have identified considerable genetic heterogeneity among strains causing mastitis. The aim of this study was to investigate the contribution of different clonal complexes and the occurrence of virulence factors and resistance determinants within bovine *Staphylococcus aureus* isolates.

A total of 189 *Staphylococcus aureus* isolates obtained from milk samples of 34 dairy herds in the German Federal State of Thuringia were characterised by microarray technology.

The isolates were assigned to eleven different clonal complexes with CC151, CC479 and CC133 being dominant (together 80.5%). The methicillin resistance gene *mecA* was found in four isolates (2.1%), which belonged to CC398. Enterotoxin genes could be detected in 79.3% of analysed *Staphylococcus aureus* and 19 isolates (10.1%) harboured a distinct allele of the toxic shock syndrome toxin gene, *tst-RF122.* The most striking finding of the present study was that almost all except one isolate (151/152) belonging to CC151, CC479 and CC133 harboured the leukocidin genes *lukF-P83* and *lukM,* whereas no further isolates from other lineages possessed these genes.

The consistent occurrence of *lukF-P83/lukM* in the dominating clonal complexes suggests an essential role of this leukocidin in the etiology of bovine mastitis.

## Background

*Staphylococcus* (*S*.) *aureus* is one of the most common etiological agents of bovine mastitis [1]. In the German Federal State of Brandenburg, *S. aureus* could be detected in 21.8% of all bacteriologically positive milk samples [2]. Similar observations were made in Belgium [3]. Some sequence types (ST) are mainly associated with cattle, whereas other strains are observed in a variety of hosts including humans [4]. Studies of the molecular epidemiology of *S. aureus* suggest that diverse strains differ in their success to cause intramammary infections in cows [1,5]. Furthermore, the carriage of different virulence factors like enterotoxins and leukocidin increases the genetic heterogeneity in mastitis-causing strains. Recently published results suggest that the leukocidin LukF-P83/LukM is involved in pathogenesis of staphylococcal mastitis [6-8]. This bicomponent leukocidin is present in the majority of *S. aureus* isolates from bovine milk samples and genes coding this leukocidin seem to be exclusively harboured by strains associated with mastitis in ruminants [9,10]. Like the human Panton-Valentine leukocidin (PVL), LukF-P83/LukM consists of two separate components S and F, which build oligomeric pores in the membrane of phagocytes and thus act as cytolytic toxins [7]. It is the most active *S. aureus* leukotoxin on bovine neutrophils [6] and may influence the severity of disease [11].

The present survey is based on a former study on the application of the microarray-technology on bovine *S. aureus* isolates [12]. The microarray used in this previous study was refined and while the focus of this first study was on sequence variations in different genes, the present investigation is focusing on the distribution and abundance of different *S. aureus* clonal complexes (CC) in 34 dairy herds in the German Federal state of Thuringia. Another aim of this present survey was to investigate the connection between CC and the virulence and antibiotic resistance gene profile of bovine *S. aureus*.

## Methods

### Isolation of *S. aureus* strains and DNA preparation

Quarter milk samples from all milk-producing cows of 34 farms were taken twice at intervals of at least eight weeks, whereas cows with clinical mastitis were excluded from sampling. In this way, 81 567 quarter milk samples from 20 838 clinically healthy cows were obtained (Table 1). Because of the dry cow period, 7476 of these cows were sampled once and 6681 cows were sampled twice. Cultivation from milk samples was done according to standard procedure [13] in the laboratory of the Livestock Health Service & Insurance Agency of the State of Thuringia. Specimens were spread on esculin blood agar (Oxoid, Wesel, Germany) and incubated at 37°C. The plates were examined after 24 and 48 h of incubation. Single colonies were picked for further subculturing. Screening for clumping factor and coagulase was performed using Staphaurex Plus (Remel, Lenexa, USA) and rabbit plasma (BioMerieux, Marcy-l’Étoile, France). To limit inclusion of *S. aureus* contaminants, only pure cultures above 1000 cfu/mL were used for further investigations. 189 out of 1902 detected *S. aureus* isolates, usually three isolates per herd sampling, were selected considering colony morphology (haemolysis, pigmentation) based on following rules:

a. In cases of unique morphology: three isolates randomly selected

b. In cases of heterogeneous morphology: one isolate of each most prevalent type.

**Table 1:** **Number of cows, proportion of*S. aureus*infected cows and frequencies of the different CC**

**Herd**	**Investigated cows**	**Proportion of*S. aureus*infected cows**	**CC151**	**CC479**	**CC133**	**CC97**	**CC398**	**CC9**	**CC20**	**CC45**	**CC101**	**CC7**	**CC50**
	**n**	**%**	**n**	**n**	**n**	**n**	**n**	**n**	**n**	**n**	**n**	**n**	**n**
1	79	50.00				6							
2	273	18.38	3						3				
3	117	33.33	6										
4	322	9.91	5	1									
5	1156	6.32	4	1			1						
6	645	3.04	1	1	4								
7	1111	6.29	1		3						2		
8	2172	13.22	1	3			2						
9	1482	2.37			3		3						
10	699	2.40		4	2								
11	349	23.21	5		1								
12	75	4.00	1	1									
13	678	0.63	1		1					1			
14	773	8.29	5	1									
15	739	6.04		3				3					
16	125	6.98	6										
17	233	8.23	3	2	1								
18	354	16.80	3	1	2								
19	684	12.27	4	2									
20	714	11.31	6										
21	259	7.88	5		1								
22	1029	3.77	6										
23	194	8.47	3	3									
24	996	0.31		1								1	
25	1738	18.89		2	2				2				
26	749	14.12	1			2		2			1		
27	32	40.00	2							4			
28	863	14.71	4	1			1						
29	74	19.51	5										1
30	410	12.27		6									
31	332	0.84	1			1							
32	380	13.65	5				1						
33	666	4.71	5		1								
34	336	12.12	6										
∑	20 838	9.19	98	33	21	9	8	5	5	5	3	1	1

Thereafter, the isolates were comprehensively characterised using microarray analysis.

An inoculation loop of *S. aureus* grown on esculin blood agar at 37°C overnight was resuspended in 300 μL of phosphate buffered saline. Genomic DNA was prepared after enzymatic lysis using lysis buffer and lysis enhancer (both from StaphyType Kit, Alere Technologies GmbH, Jena, Germany). After incubation (1 h, 37°C, 550 rpm), 40 μL proteinase K and 200 μL binding buffer (both from High Pure PCR Template Preparation Kit; Roche Diagnostics, Mannheim, Germany) were added. After a second incubation period (10 min, 72°C, 550 rpm) the samples were processed using the device of High Pure PCR Template Preparation Kit (Roche Diagnostics) according to the instructions of the manufacturer.

### Amplification, labelling, and array hybridisations

The DNA microarray of the StaphyType Kit (Alere Technologies GmbH) used in the present study covers 334 different target sequences, corresponding to, depending on the nomenclature applied, approximately 170 distinct genes and their allelic variants. It includes species markers, resistance genes, exotoxins, MSCRAMM (microbial surface components recognizing adhesive matrix molecules) genes, as well as *SCCmec*, capsule and *agr* group typing markers. The targets, related protocols, data interpretation and evaluation procedures used have been described previously [14].

An iterated linear primer elongation with one primer per target was used for the simultaneous amplification of all targets. Within this step, amplicons were labelled by incorporation of biotin-16-dUTP. After denaturation, the sample was hybridised to the array followed by washing and blocking steps. Horseradish-peroxidase-streptavidin conjugate was added. After further incubation and washing steps, hybridisations were visualised by using a precipitation dye. Finally, an image of the microarray was taken and automatically analysed using a designated reader and software (Alere Technologies GmbH).

The affiliation of isolates to CC as defined by MLST was determined by an automated software comparison of hybridisation profiles to a collection of reference strains previously characterised by MLST. The suitability of this method to assign isolates to CC was shown by former investigations [15].

### Statistical calculations

The calculation software SPSS 15.0 for Windows (Fa. SPSS Inc., Illinois, Chicago, USA) was used for statistical calculations. CC were classified according to presence of *lukF-P83/lukM*. A k*2 contingency table was tested for homogeneity of frequencies using chi-squares procedure (exact significance) and a level of significance of *p* ≤ 0.01.

### Measuring leukocidin concentration in *lukF-P83/lukM* positive isolates

To clarify if the expression of *lukF-P83/lukM* is possible at least under in vitro conditions, we measured the amount of LukF-P83/LukM in culture supernatant of 15 *lukF-P83/lukM* positive isolates as described by Monecke et al. [16].

## Results

### Species confirmation of *S. aureus*

Using microarray analysis, 189 isolates were examined for *S. aureus* specific markers like catalase (*katA*), coagulase (*coa*), protein A (*spa*) and staphylococcal accessory regulator A (*sarA*). All isolates were thus confirmed as *S. aureus*.

### Affiliation to CC

The 189 isolates were clustered into eleven different CC (Table 1). 80.5% (*n* = 152) of all isolates were assigned to CC151 (*n* = 98), CC479 (*n* = 33) and CC133 (*n* = 21). In 18 out of the 34 herds, all investigated isolates exclusively belonged to these three CC (Table 1). Furthermore, CC97 (*n* = 9), CC398 (*n* = 8), CC9 (*n* = 5), CC20 (*n* = 5), CC45 (*n* = 5), CC101 (*n* = 3), CC7 (*n* = 1) and CC50 (*n* = 1) were detected in this study.

All isolates of CC133, CC97, CC398, CC20, CC45, CC101 and CC7 belonged to *agr* group I, whereas CC151, CC479 and CC9 belonged to *agr* group II. The CC50 isolate was part of *agr* group IV.

### Leukocidin carriage

The genes encoding for Panton-Valentine leukocidin (*lukS/F-PVL*) were not found in this study, but the genes *lukF–P83* and *lukM* were detected in 151 out of 189 isolates (79.9%) belonging to CC151, CC479 and CC133 (Table 2). With the exception of one CC479 isolate, all isolates belonging to these CC possessed the genes encoding for the LukF–P83/LukM leukocidin, and none of the other CC did. Frequencies of CC with or without *lukF-P83/lukM* differed significantly (*p* < 0.01). The expressed leukotoxin could be detected in culture supernatant of all 15 tested *lukF-P83/lukM *positive isolates whereas the toxin was not detected in isolates which were genotypically negative for *lukF–P83/lukM*.

**Table 2:** Presence of genes encoding leukocidins, superantigenes, staphylokinase, as well as of resistance determinants

**CC**	**n**	***lukF-P83***	***lukM***	***lukD***	***lukE***	***tst-1***	***tst-RF122***	***sea***	***seb***	***sec***	***sel***	***egc***	***sak***	***mecA***	***blaZ***	***fosB***	***tetK***	***tetM***	***fexA***	***cat***
**CC151**	**98**	**98**	**98**	**98**	**94**	0	**19**	0	**4**	**18**	**18**	**98**	0	0	**1**	**3**	0	0	0	**1**
**CC479**	**33**	**32**	**32**	**33**	**30**	0	0	0	0	0	0	**33**	0	0	0	**2**	0	0	**2**	0
**CC133**	**21**	**21**	**21**	**21**	**2**	0	0	0	0	0	0	0	0	0	0	**20**	0	0	**3**	0
**CC97**	**9**	0	0	**9**	**7**	0	0	0	0	0	0	0	0	0	0	**2**	0	0	0	0
**CC398**	**8**	0	0	0	0	0	0	0	0	0	0	0	0	**4**	**8**	0	**3**	**7**	**3**	0
**CC9**	**5**	0	0	0	0	0	0	0	0	0	0	**5**	**1**	0	**5**	**5**	0	0	0	0
**CC20**	**5**	0	0	0	**5**	0	0	0	0	0	0	**5**	0	0	**5**	**5**	0	0	0	0
**CC45**	**5**	0	0	0	0	**1**	0	0	0	**5**	**5**	**5**	**1**	0	**1**	0	0	0	0	0
**CC101**	**3**	0	0	**3**	**3**	0	0	0	0	0	0	0	**3**	0	0	**3**	0	0	0	0
**CC7**	**1**	0	0	**1**	**1**	0	0	**1**	0	0	0	0	**1**	0	**1**	0	0	0	0	0
**CC50**	**1**	0	0	**1**	**1**	0	0	0	0	0	0	**1**	0	0	0	0	0	0	0	0
∑	**189**	**151**	**151**	**166**	**143**	**1**	**19**	**1**	**4**	**23**	**23**	**147**	**6**	**0**	**21**	**40**	**3**	**7**	**8**	**1**

The genes encoding the leukocidin LukD/E were detected in all isolates belonging to CC7, CC20, CC50, CC97, CC101, CC133, CC151 and CC479, although some of these isolates gave variable, weak or negative results for either *lukD* or *lukE* (data not shown). This might be regarded as an indicator for sequence variations. Leukocidin genes *lukD* and *lukE* were not found in isolates belonging to CC398, CC9 and CC45 (Table 2).

### Superantigen carriage

Nineteen out of 189 isolates affiliated to CC151 harboured a distinct allele of the toxic shock syndrome toxin gene as known from the genome sequence of strain RF122 [GenBank: AJ938182], designated here as *tst-RF122* (Table 2)*.* Another allele of *tst-1,* as observed in isolates from humans (such as in BA000017.4), was present in only one sample belonging to CC45.

Enterotoxin genes were found in 78.3% of analysed isolates. Genes of the *egc*-enterotoxin gene cluster (*seg* + *sei* + *sem* + *sen* + *seo* + *seu/y*) were found most frequently, in 147 isolates belonging to CC9, CC20, CC45, CC50, CC151 and CC479.

The genes encoding for the classical enterotoxins A-C (*sea, seb, sec*) were comparatively rare (Table 2). The enterotoxin A gene *sea* was only found in the single CC7 isolate, being present in an allele as previously described from the CC5 strain N315. Twenty-three out of 189 isolates harboured *sec* and *sel,* which are commonly located on pathogenicity island SaPIbov [17]. All *sec/sel* positive isolates belonging to CC151 (*n* = 18) also harboured *tst-RF122. seb* was detected in four isolates combined with *sec*. The genes encoding enterotoxin D, E, H, J, K, Q and R were not found among isolates tested.

### Other virulence genes

The gene encoding staphylokinase (*sak*) was only detected in all three isolates belonging to CC101 and one isolate of each CC9, CC45, and CC7, representing 3.2% of all samples (Table 2).

The majority of isolates (89.9%) possessed the haemolysin beta gene *hlb*. Genes for haemolysins alpha, delta, and gamma (*hla*, *hld*, and the *hlg*-locus comprised of *lukS*, *lukF*, and *hlgA*) were present in all isolates.

Exfoliative toxin genes (*etA, etB, etD*) and epidermal cell differentiation inhibitor genes (*edinA-C*) were not detected.

### Antibiotic resistance genes

The methicillin resistance gene *mecA* was found in four isolates (2.1%), which all belonged to CC398 (Table 2). They also carried the β-lactamase operon *blaZ*, *blaI* and *blaR* and the tetracycline resistance determinant *tetM*. The gene *tetK,* which also confers resistance to tetracyclines, was detected in three *mecA*-positive CC398 isolates. Among the other isolates, resistance genes were generally rare. Beside the CC398-MRSA isolates, seventeen further isolates harboured the β-lactamase gene *blaZ*. The gene was detected in all isolates belonging to CC9, CC20 and CC398 (Table 2). Moreover, *blaZ* was found in one isolate of each CC151, CC45 and CC7. The gene *tetM* was found in three methicillin-susceptible CC398 isolates. Thirty-nine methicillin susceptible *S. aureus* (MSSA) isolates harboured the putative fosfomycin/bleomycin resistance gene *fosB.* The chloramphenicol resistance determinant *fexA* was found eight times (Table 2); one CC151 isolate contained another chloramphenicol resistance determinant, *cat*. Another CC151 isolate harboured the macrolide resistance determinant *msrA.* The streptogramin resistance gene *vgaA* and *aacA-aphD,* which confers resistance to aminoglycosides gentamicin and tobramycin, were detected in one CC398 isolate each. None of the other resistance genes represented on the array was found.

## Discussion and conclusions

Previously conducted studies in the USA, the UK, Norway, Switzerland and Chile showed that only a few *S. aureus* genotypes cause the majority of bovine mastitis cases [1,18-21]. In our study, CC151, CC479 and CC133 were the dominating CC. In 18 out of 34 participating herds, only these three CC could be detected, while only in one dairy herd (herd 1) none of these CC was found (Table 1). In this herd of 45 lactating cows, which is an exceptionally small one compared to the other herds, the proportion of *S. aureus*-infected cows was very high (50%) and all six tested isolates belonged to CC97, which is a CC known to occur in ungulates.

According to the current state of knowledge, no CC151, CC479 and CC133 isolates were found in humans up to now. While CC151 and CC479 are restricted to cattle [4,9,21], CC133 also occurs in sheep and goats [22]. *S. aureus* belonging to the other CC, which were only sporadically found in milk samples, are not restricted to a particular host species. CC7, CC9, CC20, CC45 and CC101 were preferentially isolated from human sources [23]. However, their occurrence is also described for different pets and livestock [12,24,25]. CC50 was equally detected in animals [4] as well as in human samples [26]. CC97 is quite frequently found in bovine mastitis, but it can also be detected in human cases [21]. CC398 is of outstanding importance in livestock and can be found in a variety of animal species including poultry, swine, horses and cattle [12,27-29]. Methicillin resistant strains of this CC (CC398-MRSA) have recently received a lot of attention as strains from this lineage are regarded as zoonotic [30,31]. Four *mecA*-positive strains in this study belonged to CC398 (2.1% of all tested isolates). Compared with results obtained from German pig herds with MRSA prevalences up to 70.8% [31-33], the detected prevalence in our cattle study seems low. Our results are in accordance to a previous study reporting a MRSA prevalence of 1.6% among bovine isolates from Germany and Switzerland [12]. Similar low prevalences of MRSA in the context of bovine mastitis have also been reported from other countries [34,35].

Apart from the *mecA*-positive isolates, resistance genes were rare. Twenty-one isolates carried *blaZ* (11.1%), whereas 20 of these isolates belonged to strains without a distinct host species specificity. Forty isolates (21.2%) harboured *fosB*. The tetracycline resistance determinant *tetM* was detected in seven out of eight CC398 isolates (4 × MRSA, 3 × MSSA). Three *tetM*-positive MRSA isolates additionally possessed *tetK*. The simultaneous presence of *tetK* and *tetM* is more prevalent in MRSA isolates than in MSSA isolates and the strains harbouring both genes displayed higher MIC values than those carrying just one of the genes [36]. The increased resistance to tetracycline seems to be a selective advantage.

The low occurrence of resistance genes in bovine isolates could indicate a lower selective pressure in consequence of a more constricted use of antibiotics in bovine medicine contrary to human medicine. It has to be mentioned that array targets are designed for human isolates. Therefore, certain resistance markers such as *ermT, dfrK, tetL* and *vgaE* might have remained undetected.

The staphylokinase gene *sak* was found only sporadically in our study. None of the CC151, CC479 and CC133 isolates carried *sak*. In contrast, undisrupted *hlb* was found in the majority of isolates. Similar results were reported by Sung et al. [21]. In human strains, *sak* is highly prevalent [37] and *hlb* is usually inactivated by the insertion of phages carrying s*ea* as well as *sakscn* and *chp* in various combinations [38]. The latter authors speculated that the presence of *hlb,* which encodes a phospholipase C, in bovine mastitis provides a higher selective advantage than staphylokinase. The simultaneous occurrence of *sak* and undisrupted *hlb* in five strains could possibly indicate the integration of a phage at an unusual site in the *S. aureus* genome or the presence of mixed populations in which some cells carry the phage while others lack it. One *sak* and *hlb* positive isolate also carried *sea*. Because of its typical occurrence in human strains, the presence of *sak* could possibly indicate a human (milker)-to-cow transmission. Particularly with regard to one CC45 isolate in our investigations, such a transfer appears to be probable. This one did not possess undisrupted *hlb*, but carried *sak* and an allele of *tst-1* usually associated with human strains, a combination not found in any other isolate. It cannot be completely excluded that the source of this CC45 isolate was a contamination. However, this source seems unlikely because only pure cultures above 1000 cfu/mL were used for further investigations.

The most striking finding of the present study was that almost all isolates belonging to CC151, CC479 and CC133 harboured the combination of *lukM* and *lukF-P83* while no isolates from the other CC carried these genes. The high level of significance between CC and the presence of this leukocidin indicates a high clinical or epidemiological relevance of this factor. LukM and LukF-P83 are components of a leukocidin originally described in bovine *S. aureus* strain P83. The corresponding genes *lukM* and *lukF-P83* lie on the genome of a prophage and are encoded as one operon similar to that of the Panton-Valentine leukocidin [7]. Based on our investigations, the presence of *lukF-P83/lukM* is strictly associated with certain *S. aureus* lineages. According to the current state of knowledge these lineages are limited to cattle and small ruminants [4,9,21,22].

In recent years, some authors referred to the heterogeneity of different *S. aureus* strains in epidemiology, prognosis and therapy [20,39]. While some strains - comparable to *Streptococcus agalactiae* - behave very contagiously, other strains show the nature of environmental pathogens and can also be detected outside the cows in the stable [40]. Because of the host specificity of certain CC from cattle, other hosts (e.g. milkers, yard dogs or rodents) probably cannot serve as a reservoir. The detection of *lukF-P83/lukM* in selected isolates of a dairy farm could facilitate an identification of cattle-adapted *S. aureus* strains, which can more easily be targeted by infection control programs than strains originating from other sources. Furthermore, the consistent occurrence of *lukF-P83/lukM* in the dominating CC hypothesises an essential role of this leukocidin on the etiology in bovine mastitis. Up to now, only in vitro investigations regarding the pathogenicity of LukF-P83/LukM have been made. Rainard et al. [8] investigated the leukotoxic potential of different *S. aureus* strains towards ruminant neutrophils. According to this study, *lukF-P83/lukM*-positive strains had a significantly higher leukotoxicity than strains without these genes.

In the bovine udder, the presence of these leukocidin genes in *S. aureus* could be a crucial selective advantage. A leukotoxicity, provided it also exists in vivo, could lead to reduction of host defense and facilitate more rapid colonisation of the bovine udder by pathogens. In addition, the potentially mobile localisation of *lukF-P83/lukM* on the genome of a prophage [7] might also indicate a relevance of the leukocidin for *S. aureus* in a bovine environment. If a prophage is consistently present in a host population it should be expected that its presence confers some evolutionary advantages. If its integration might cause a disadvantage such as slower replication, *S. aureus* populations without this phage should prevail. If it causes neither advantage nor disadvantage, it should be rather rare as acquisitions as well as loss are random events.

One CC479 isolate did not possess *lukF-P83/lukM*. This indicates that loss of the prophage from an isolate belonging to one of the dominating CC in the study still might occur. The particular CC479 isolate without *lukF-P83/lukM* was apparently unable to spread during the study period as subsequently, no further *S. aureus* was identified in this herd.

From these observations, analogies to PVL may be drawn. PVL positive *S. aureus* strains have spread worldwide in recent years [37]. While infections caused by PVL negative *S. aureus* strains in persons without health-care-associated risk factors are often asymptomatic, infections with PVL positive strains usually cause severe symptoms in otherwise healthy children and young adults such as recurrent/chronic skin and soft tissue infections or necrotising pneumonia [41]. Similar to PVL, LukF-P83/LukM could determine whether *S. aureus* behaves as an accidental or as an obligatory pathogen in its respective host organism. LukF-P83/LukM negative strains would then be accidental strains in cattle, possibly being transferred from other species such as humans or rodents into cows. LukF-P83/LukM positive strains could then be regarded as obligatory pathogens to cattle which also implied that they might easily spread and persist in herds. For surveillance a rapid “Point of Care” test for this virulence marker would be a helpful tool.

In conclusion, the strikingly high prevalence of CC harbouring *lukF-P83/lukM* leads to the hypothesis, that strains with these genes possess advantages capable to dominate in the spread of the infection within the herd. Although we yet lack the understanding of the underlying mechanism of this selection, we assume that the occurrence of *lukF-P83/lukM* might have an influence on the severity of bovine mastitis. However, this has to be subject of further investigations. Beyond that, it will be necessary to investigate the geographic distribution and the pathogenic properties of CC of *S. aureus* in other regions, in order to see whether or not the sole detection of *lukF-P83/lukM* would be suitable for mastitis-causing *S. aureus*.

## Competing interests

The authors declare that they have no competing interests.

## Authors’ contributions

KD and HH conceived the study, and developed its design and coordination. MP, RE, SM and KS participated in study design. KS carried out the bacteriological investigations, DNA preparation and hybridisation. The expression of leukocidin was tested by RE. KD and KS carried out the statistical calculations. The manuscript was drafted by KS and was revised critically by RE, HH, SM, MP, and KD. All authors read and approved the final manuscript.
